# Supplementary material for: Hexokinase 2-driven glycolysis in pericytes activates their contractility leading to tumor blood vessel abnormalities
Source: Nat Commun. 2021 Oct 14;12:6011. doi: 10.1038/s41467-021-26259-y (PMC8517026; doi:10.1038/s41467-021-26259-y)
Supplement: Supplementary file 1 — Supplementary Information [file 41467_2021_26259_MOESM1_ESM.pdf]

Supplementary Information

Hexokinase 2-driven glycolysis in pericytes activates their contractility leading to tumor blood vessel abnormalities

Ya-Ming Meng[1,2,3#], Xue Jiang[1,3,#], Xinbao Zhao[1,4,#], Qiong Meng[1,3], Sangqing Wu[1,3], Yitian Chen[1,3], Xiangzhan Kong[1,3], Xiaoyi Qiu[1,3], Liangping Su[1,3], Cheng Huang[1,3], Minghui Wang[5], Chao Liu[6] and Ping-Pui Wong[1,3*]

Supplementary Table 1. Sequences of Primers

Name	Sense Strand (5'-3')	Antisense Strand (5'-3')
CD31	CGTCAAGCCTCAGCACCAGATG	GCACTCCTTCCACCAACACCTG
CD34	ACCAGAGCTATTCCCAAAAGACC	TGCGGCGATTCATCAGGAAAT
CD45	ACAGCCAGCACCTTTCCTAC	GTGCAGGTAAGGCAGCAGA
CNN1	CAACCACCACGCACACAACTAC	GGTCCAGCCAAGAGCAGCAG
MYCOD	CTCGGCTTCCTTTGAACAAG	CTTCCCAGAGAATCCATCCA
ACTA2	CTCTGGACGCACAACTGGCATC	CGGACAATCTCACGCTCAGCAG
FAP	TGGTGGATGGTCGAGGAACAGC	TGCAAGGGCCAGTGATGAAACG
PDGFRA	TTGAAGGCAGGCACATTTACA	GCGACAAGGTATAATGGCAGAAT
PDGFRB	TGCAGCACCACTCCGACAAGC	TCGCTCTCCCCGGTCAAGGAC

CD146	GAAGTCACCGTCCCTGTTTTC	CCCCGTTGTCGTTGGTTGT
GLUT1	ATTGGCTCCGGTATCGTCAAC	GCTCAGATAGGACATCCAGGGTA
GLUT2	TGTGCGAGCCATCCTTCAGTCT	TGAGCCACATGCAGCATCAGTG
HK1	CACATGGAGTCCGAGGTTTATG	CGTGAATCCCACAGGTAACTTC
HK2	AACAGCCTGGACGAGAGCAT	GCCAACAATGAGGCCAACTT
GPI	GATGGTAGCTCTCTGCAGCC	GCCATGGCGGGACTCTTG
PFKFB3	GTGCGACGACCCTACAGTTGTG	AGTACACGATGCGGCTCTGGAT
PFKM	CCAGCCTGTGTAGTGAGCCTCT	CAGTGGAGCGAACAGCAGCATT
PFKL	GCTGGGCGGCACTATCATT	TCAGGTGCGAGTAGGTCCG
PFKP	GACCTTCGTTCTGGAGGTGAT	CACGGTTCTCCGAGAGTTTG
ALDOA	ATGCCCTACCAATATCCAGCA	GCTCCCAGTGGACTCATCTG
ALDOB	TGTCTGGTGGCATGAGTGAAG	GGCCCGTCCATAAGAGAAACTT
GAPDH	GAGTCAACGGATTTGGTCGT	GACAAGCTTCCCGTTCTCAG

PGK1	CGTTGACCGAATCACCGACCTC	ACCGACTTGGCTCCATTGTCCA
PGK2	AGGCGTTAGAAGTCACCACCGA	TGGCTCCATTGTCCAGGCAGTA
PGAM1	TGGAACCTGGAGAACCGCTTCA	TCACCACTGGCAGCCACATCT
PGAM2	AGAAGCACCCCTACTACAACTC	TCTGGGGAACAATCTCCTCGT
ENO1	GCCGTGAACGAGAAGTCCTG	ACGCCTGAAGAGACTCGGT
ENO3	TATCGCAATGGGAAGTACGATCT	AAGCTCTTATACAGCTCTCCGA
PKM2	ACGAGAACATCCTGTGGCTG	AGGAAGTCGGCACCTTTCTG
PKLR	CCTGTGGAAGCGGTGAAGATGC	ACCAATGGCGGTGACCTCAGT
LDHA	TTGACCTACGTGGCTTGGAAG	GGTAACGGAATCGGGCTGAAT
LDHB	TCTGTGACCGCCAATTCTAAGA	GCACCAGATTGAGCCGACTC
ROCK1	GGTGGTCGGTTGGGGTATTTT	CGCCCTAACCTCACTTCCC
ROCK2	TGGTTTCTATGGGCGAGAATGT	CAAGTCGTACCTCCCTATCTGTT

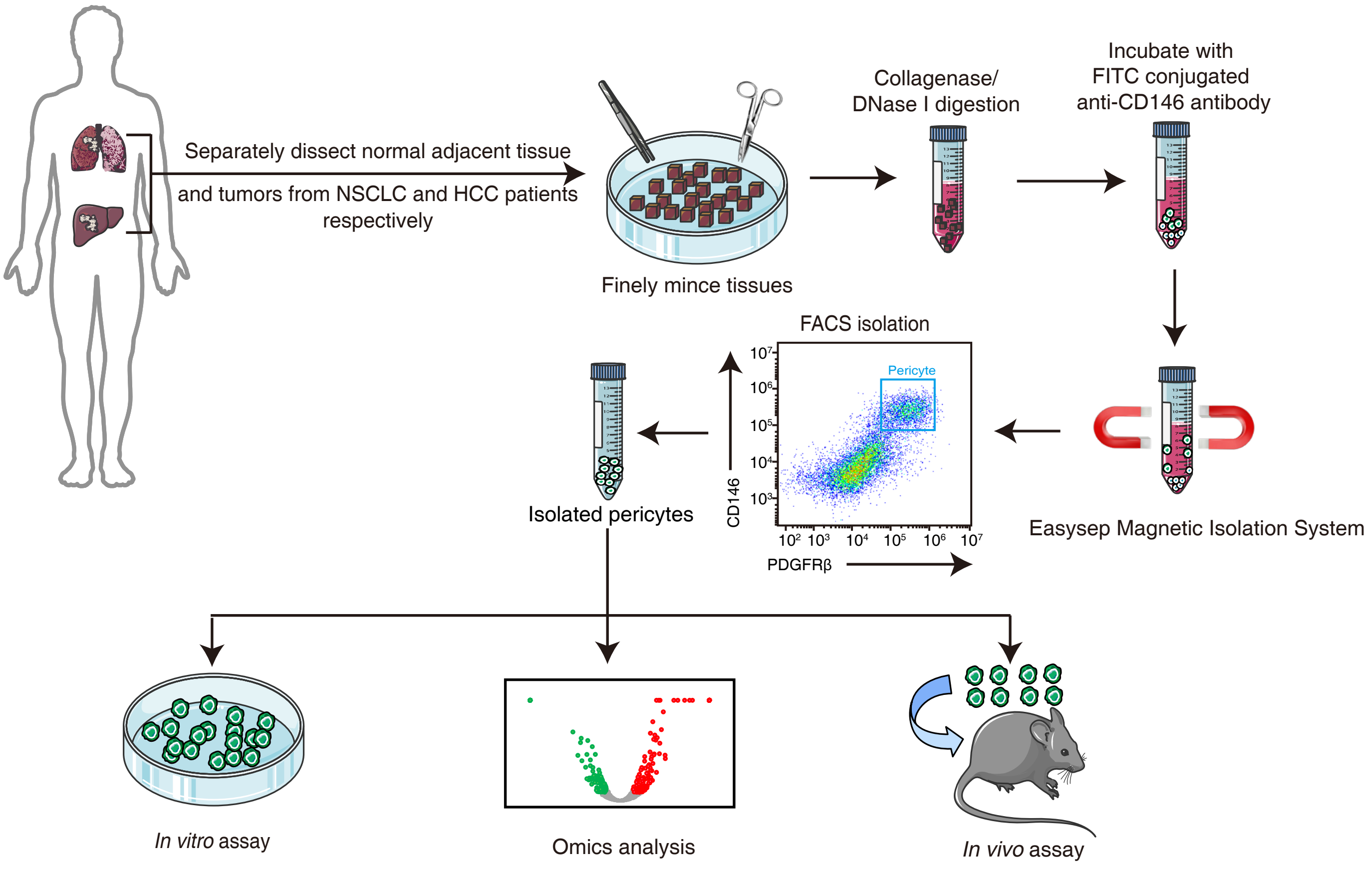
Separately dissect normal adjacent tissue
and tumors from NSCLC and HCC patients respectively
Finely mince tissues
Collagenase/ DNase I digestion
Incubate with FITC conjugated anti-CD146 antibody
Easysep Magnetic Isolation System
FACS isolation
Pericyte
CD146
PDGFRβ
Isolated pericytes
In vitro assay
Omics analysis
In vivo assay

Supplementary fig. 1: Schematic diagram of purifying primary pericyte population from normal adjacent tissues and tumors derived from NSCLC and HCC patients. Solid tumors and normal adjacent tissues derived from NSCLC or HCC patients were finely minced with surgical scissors. The minced tissues were then digested with collagenase and DNase I, and the undigested tissue mass fragment and debris were eliminated by different size strainer and erythrocytes eliminated by RBC lysis buffer. The single-cell suspension was first sorted by using a $CD146^{+}$-FITC-microbead activated cell soring (MACS) method to enrich vascular cell population, which was then subjected to FACS using pericyte positive and negative selection markers. The isolated pericytes could be used for omics analysis *in vitro* and *in vivo* assays. Parts of the figure are drawn by using pictures from Servier Medical Art. Servier Medical Art by Servier is licensed under a Creative Commons Attribution 3.0 Unported License (https://creativecommons.org/licenses/by/3.0/).

Supplementary figure 2

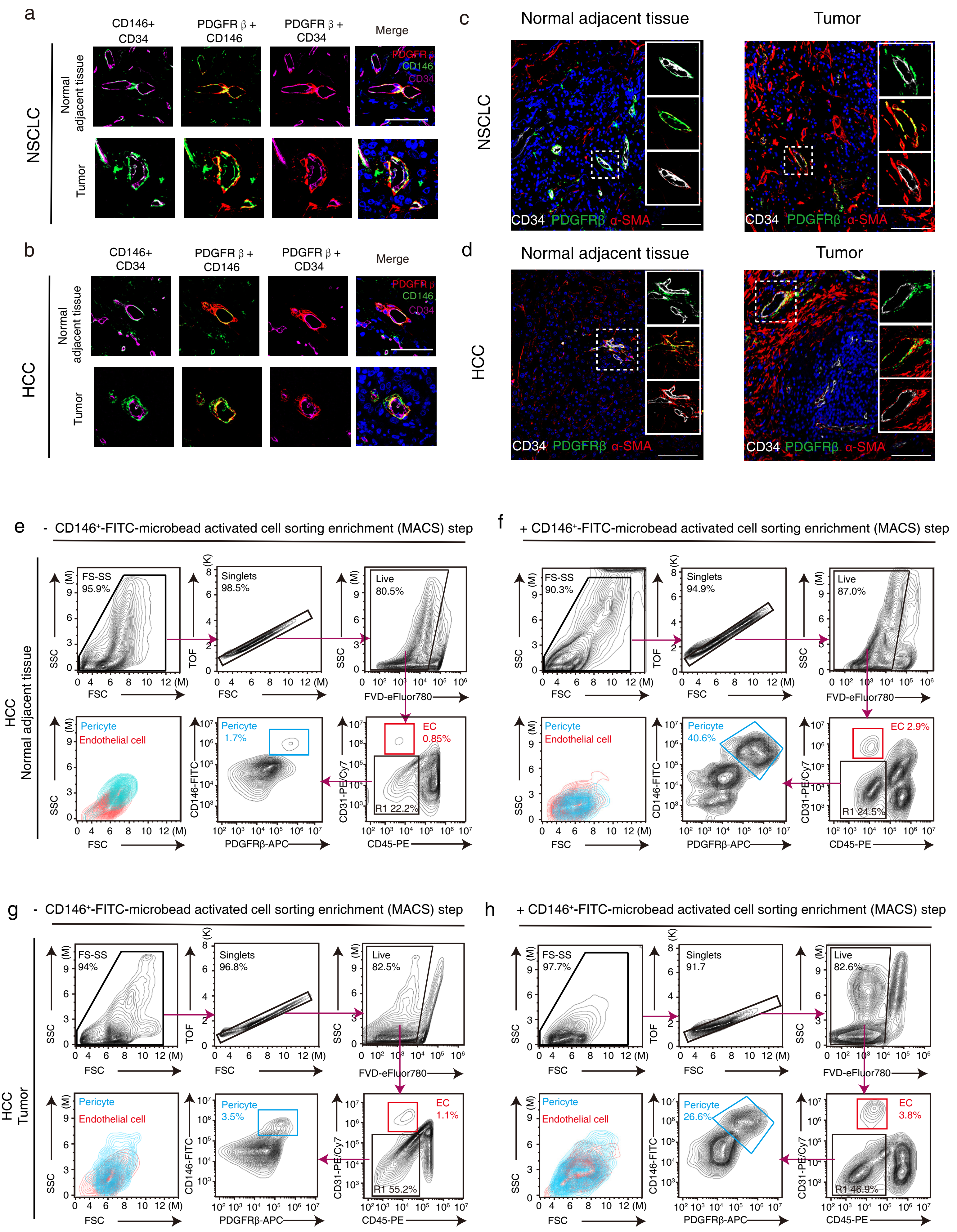

Supplementary fig. 2: FACS gating strategy for Isolating pericytes from normal adjacent tissues and tumors derived from HCC patients. (a-d) Triple immunostaining of CD34 (magenta)/CD146 (Green)/PDGFRβ (red) or CD34 (white)/PDGFRβ (green)/α-SMA (red) in normal adjacent tissues and tumors derived from NSCLC and HCC patients (n=3 independent experiments). (e-h) Representative FACS plots showing the gating strategy for isolation of pericytes from normal adjacent tissues and tumors derived from HCC patients. After excluding debris (top left), non-singlets (top right) and dead cells (FVD, center right), $CD31^{+}CD45^{-}$ cells are endothelial cell (center left), $CD31^{-}CD45^{-}CD146^{+}PDGFR\beta^{+}$ cells are pericytes (center left and bottom left), the cell size and granularity of pericytes and endothelial cells are shown in bottom left. MACS, microbead activated cell sorting; FACS, fluorescence activated cell sorting; FSC, forward-scatter; SSC, side-scatter; TOF, time of fly; FVD, fixable viability dye. Scale bars in (a, b) represent 20 μm, (c, d) 50 μm, (c, d (magnified pictures)) 25 μm.

Supplementary figure 3

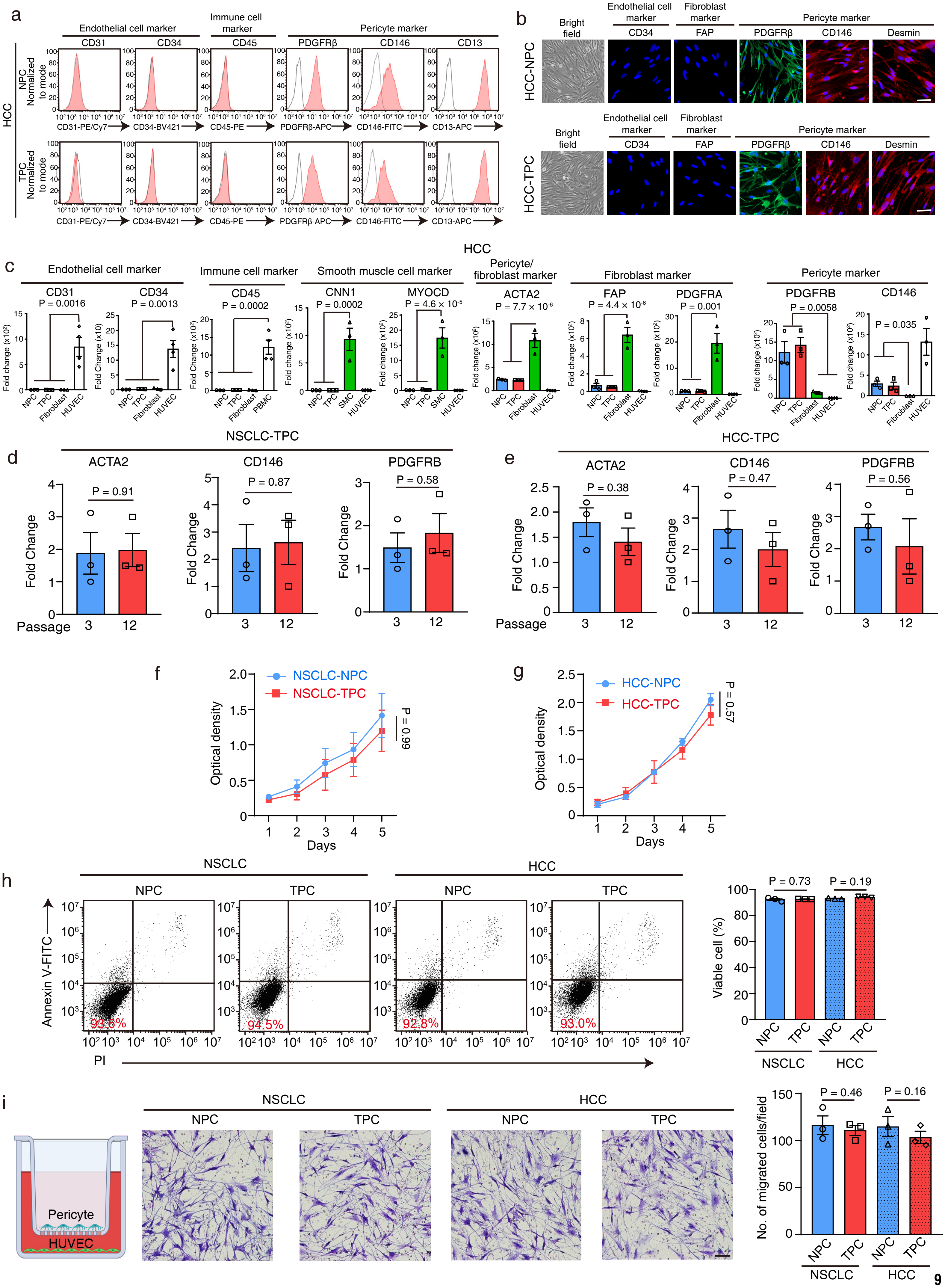

Supplementary fig. 3: Characterization and functional tests confirms the pericyte identity of our isolated cells. (a) Flow cytometry analysis of pericytes isolated from normal adjacent tissue and tumor derived from HCC patients showed that the purity of pericyte preparation was high. Pericyte preparations displayed good expression of pericyte markers PDGFRβ, CD146 and CD13 and undetected or low level of endothelial cell markers CD31 and CD34 as well as immune cell marker CD45 (n=3 individual patients). (b) Immunofluorescent staining analysis of the endothelial cell, fibroblast, pericyte marker expression in HCC derived-NPC and-TPC (n=3 independent experiments). (c) RT-PCR analysis of the endothelial cell, fibroblast, smooth muscle cells and pericyte marker expression in HCC derived-NPC and-TPC as well as HUVEC, fibroblast, peripheral blood mononuclear cells (PBMC), smooth muscle cells (SMC) (for *CD31, CD34, CD45, CNN1, MYOCD, ACTA2, FAP, PDGFRA* and *PDGFRB* expression analysis: n=3 independent NPC, TPC, Fibroblast and SMC samples and n=4 independent HUVEC and PBMC samples; for *CD146* expression analysis: n=3 independent samples for each cell type). (d, e) RT-PCR analysis of the pericyte marker expression in NSCLC/HCC derived TPC at different passage (n=3 independent experiments). Statistical tests were two-sided. (f, g) CCK8 proliferation assay showed no significant difference in the proliferation between NSCLC/HCC derived-NPC and-TPC (n=3 independent experiments). (h) Cell viability assay (PI/annexin V) of NSCLC/HCC derived-NPC and-TPC. Bar chart shows the percentage of viable cells in each group (n=3 independent experiments). Statistical tests were two-sided. (i) Schematic diagram showed that NSCLC/HCC derived-NPC/TPC were plated onto the upper compartment of a transwell and HUVEC placed onto the bottom compartment. No significant difference in the endothelial cell dependent recruitment/migration between NSCLC/HCC derived NPC and TPC (n=3 independent experiments). Statistical tests were two-sided. Results are given as means ± SEM. (c) One-way ANOVA. (d, e, h, i) Student's t test. (f, g) Two-way ANOVA. Scale bars in (b) represents 50 μm, (i) 100 μm.

Supplementary figure 4

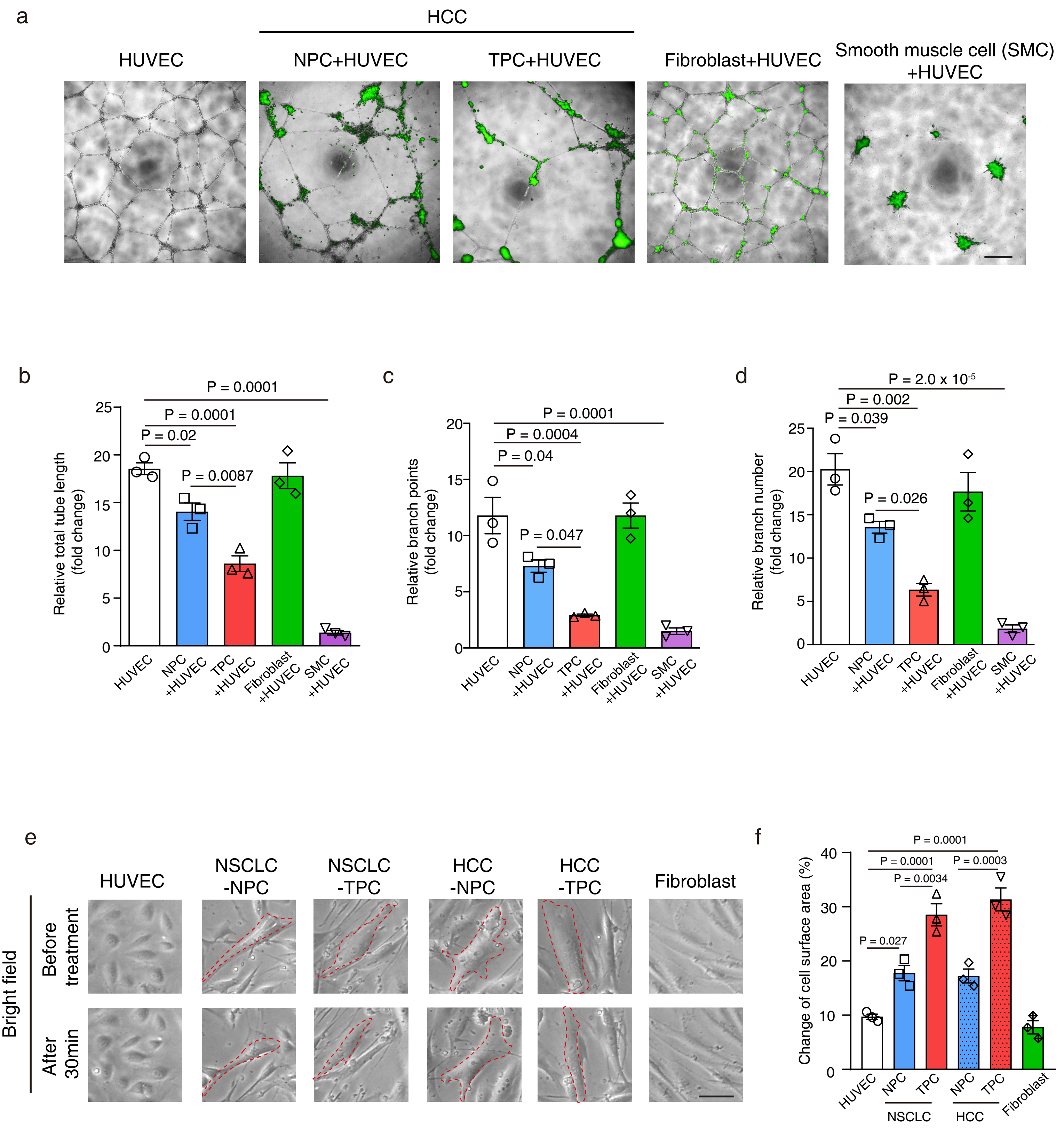

Supplementary fig. 4: Tumor derived pericytes display abnormal blood vessel supporting functions and enhanced cell contractility as compared with normal adjacent tissue derived pericytes. (a) HUVEC was cultured alone or co-cultured with CFSE labeled HCC derived NPC/TPC, fibroblast or smooth muscle cell (SMC) in a 3D Matrigel. (b-d) Bar charts show the relative total tube length, branch points and number in each group (n=3 independent experiments). (e) Representative bright field images of NPC and TPC before and after 1 μM carbachol treatment. Red lines indicate the change in surface area of NSCLC/HCC derived-NPC or -TPC before and after carbachol treatment. (f) Bar chart shows the percentage of change of cell surface area in each group (n=3 independent experiments). Results are given as means ± SEM. (b-d, f) One-way ANOVA. Scale bars in (a) represents 200 μm, (e) 50 μm.

Supplementary figure 5

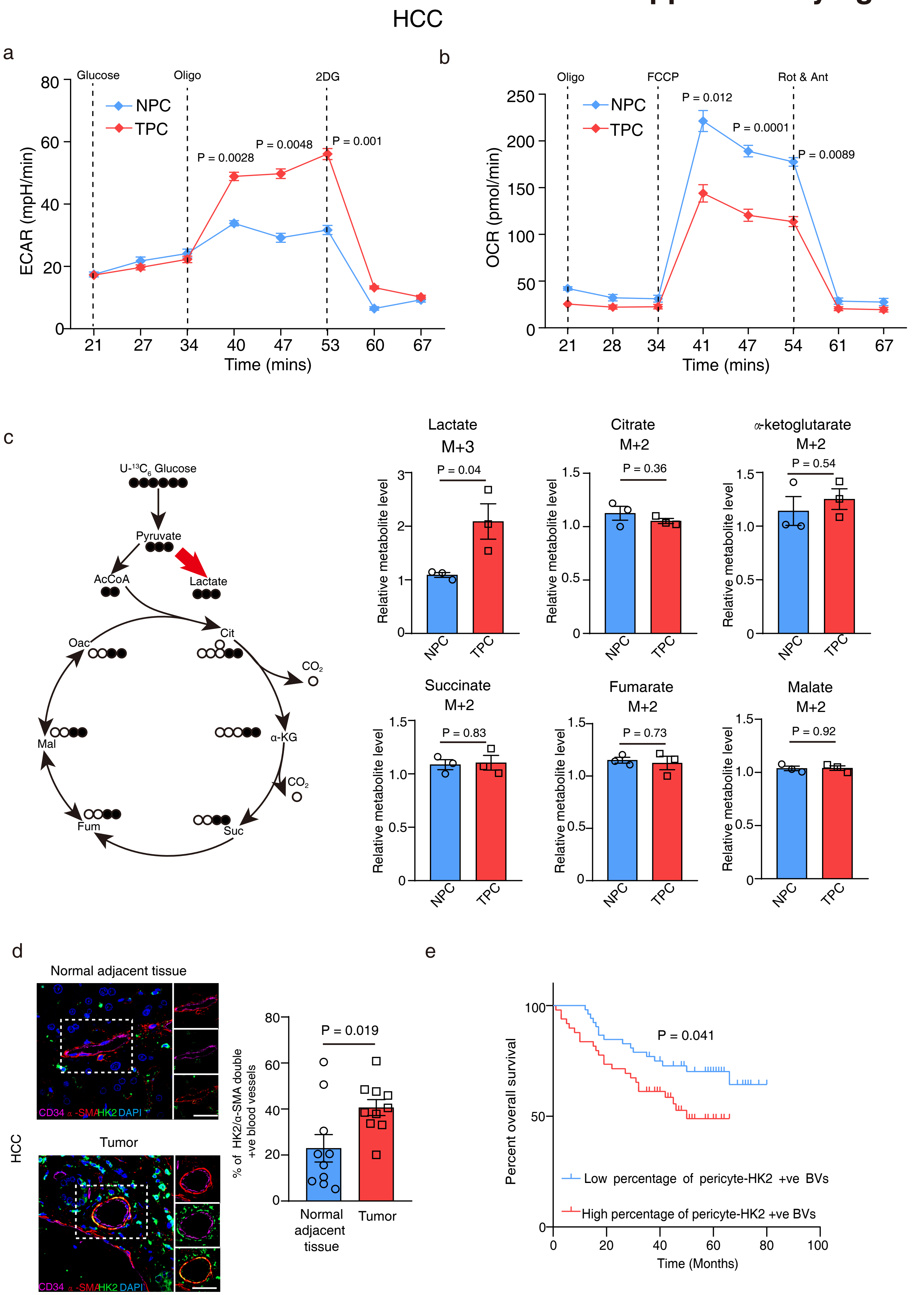

Supplementary fig. 5: Metabolic study shows an elevated glycolysis in HCC derived TPC as compared with NPC. (a, b) The extracellular acidification rate (ECAR) and oxygen consumption rate (OCR) of HCC derived NPC/TPC was measured by using a Seahorse flux analyzer (n=5 independent experiments). Dot lines indicate the time and period of glucose, oligomycin (Oligo), 2-Deoxy-D-glucose (2-DG), carbonyl cyanide-p-trifluoromethoxyphenylhydrazone (FCCP), rotenone (ROT) or antimycin (Ant) being added during seahorse experiments. Statistical tests were two-sided. (c) Metabolic flux analysis showed that the relative abundance of lactate, but not other TCA cycle related metabolites, was up-regulated in HCC derived TPC as compared to NPC (n=3 independent samples). Statistical tests were two-sided. (d) Triple immunostaining of CD34 (magenta), HK2 (green) and α-SMA (red) in normal adjacent tissues and tumors derived from HCC patients. Bar chart shows the percentage of HK2 and α-SMA double positive blood vessels in each group (n=10 HCC patients). Statistical tests were two-sided. (e) High percentage of pericyte-HK2 positive blood vessels associated with poor overall survival in HCC patients (n=101 HCC patients). Statistical tests were two-sided. (a-d) Student's t test. (e) Log-rank (Mantel-Cox) test. Scale bars in (d) represents 15 µm.

Supplementary figure 6

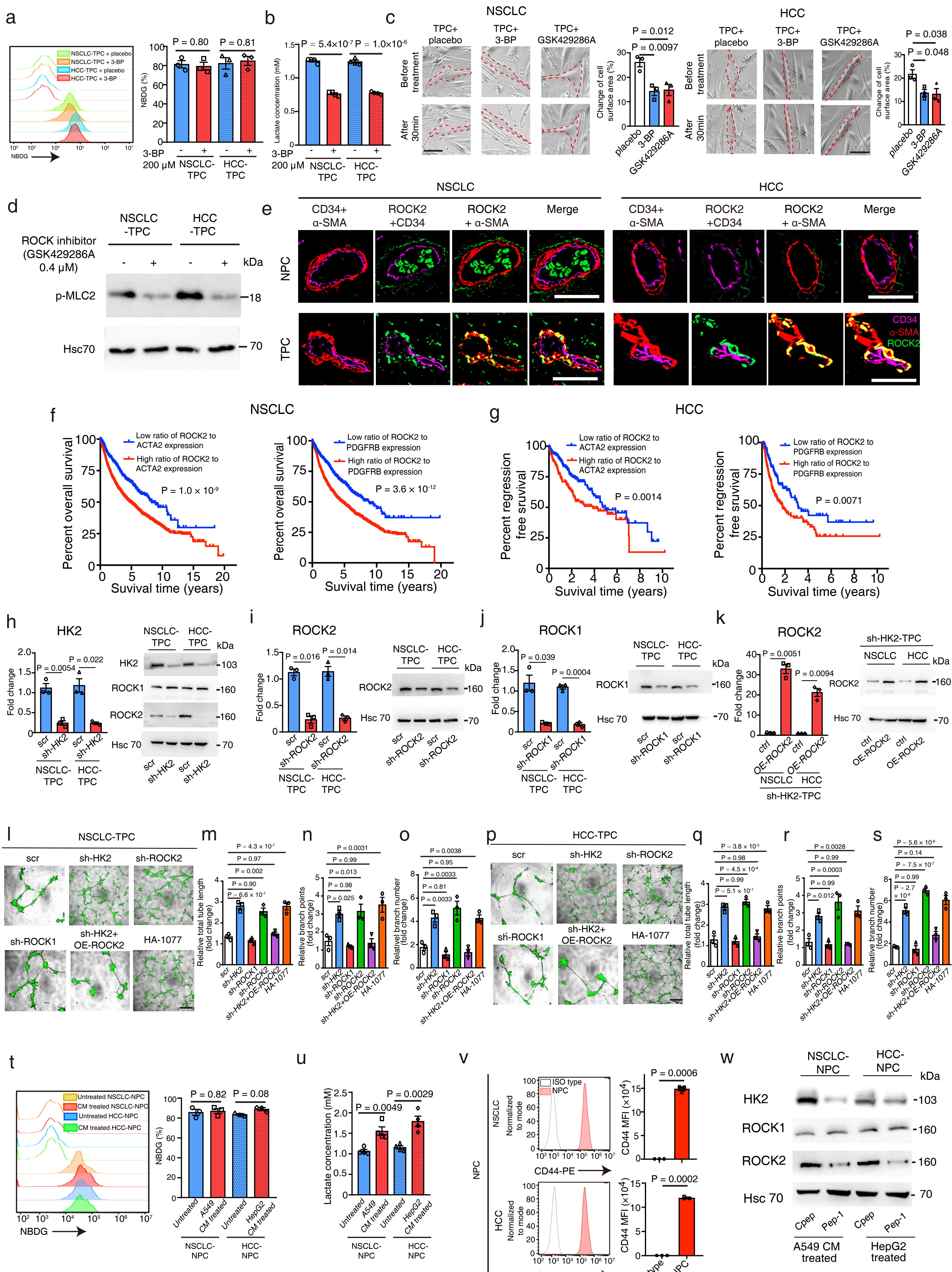

Supplementary fig. 6: Tumor derived pericytes upregulate HK2-driven glycolysis to induce ROCK2-MLC2 mediated contractility. (a) Relative glucose uptake of NSCLC/HCC derived TPC after pre-treated with or without 3-bromopyruvate (3-BP) for 15 mins (n=3 experimental repeats). Statistical tests were two-sided. (b) Pre-treatment with HK2 inhibitor 3-BP reduced lactate production in TPC (n=4 experimental repeats). Statistical tests were two-sided. (c) Pre-treatment with 3-BP/GSK429286A prohibited carbachol induced cell contraction in the indicated groups (n=3 independent experiments). Statistical tests were two-sided. (d) Western blotting analysis of the p-MLC2 expression in TPC treated with ROCK inhibitor (GSK 429286A) or placebo (n=3 independent experiments). (e) Representative images of the triple immunostaining of CD34 (magenta), ROCK2 (green) and α–SMA (red) in normal adjacent tissues and tumors (n=3 independent experiments). (f, g) High ratio of ROCK2 to ACTA2/PDGFRB expression correlated with poor overall/regression free survival in patients (n=1925 NSCLC patients, n=316 HCC patients (right panel), n=364 HCC patients (left panel)). Statistical tests were two-sided. (h-k) RT-PCR and western blotting analysis of the HK2, ROCK1 and/or ROCK2 expression in the indicated groups (n=3 independent experiments). Statistical tests were two-sided. (l-s) HUVEC were co-cultured with indicated CFSE labelled NSCLC/HCC derived TPC in a tube formation assay. Bar charts show the relative total tube length, branch points and number in each group (n=3 independent experiments). (t) Glucose uptake assay in A549/HepG2 conditioned medium (CM) treated NPC as compared with untreated NPC (n=3 independent experiments). Statistical tests were two-sided. (u) Lactate production in A549/HepG2 conditioned medium treated NPC as compared with untreated NPC (n=3 independent experiments). Statistical tests were two-sided. (v) FACS analysis of the CD44 expression on NSCLC/HCC derived NPC. Statistical tests were two-sided. (w) Western blotting analysis of the HK2, ROCK1 and ROCK2 expression in NPC after treated with conditioned medium in the presence of either HA antagonist (Pep-1) or control peptide

(Cpep). Hsc70 was used as a loading control (n=3 independent experiments). Results are given as means ± SEM. (a-b, h-k, t-v) Student's t test. (c, m-o, q-s) One-way ANOVA. (f-g) Log-rank (Mantel-Cox) test. Scale bars in (c) represents 50 µm, (e) 20 µm, (l, p) 200 µm.

Supplementary figure 7

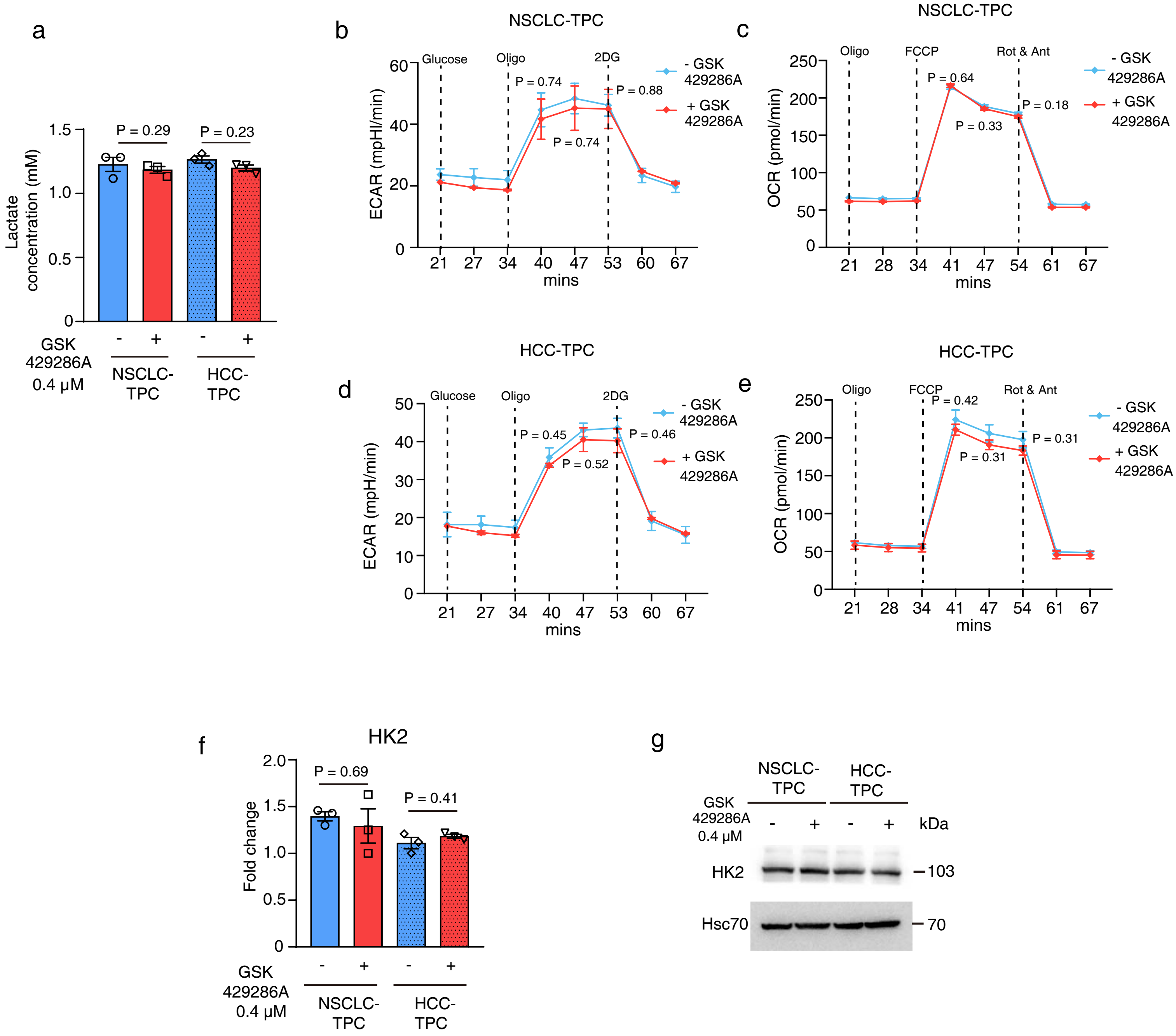

Supplementary fig. 7: Administration of ROCK inhibitor does not affect cell metabolism and HK2 expression in TPC as compared with placebo treated group. (a) Lactate concentration measurement assays of the NSCLC/HCC derived TPC after pre-treated with or without ROCK inhibitor. Statistical tests were two-sided. (b-e) Seahorse assays show the extracellular acidification rate (ECAR) and oxygen consumption rate (OCR) of NSCLC/HCC derived TPC after pre-treated with or without ROCK inhibitor GSK429286A. Dot lines indicate the time and period of glucose, oligomycin (Oligo), 2-Deoxy-D-glucose (2-DG), carbonyl cyanide-p-trifluoromethoxyphenylhydrazone (FCCP), rotenone (ROT), or antimycin (Ant) being added during seahorse experiments. Statistical tests were two-sided. (f, g) RT-PCR and Western blot analysis of the HK2 expression in TPC after treated with or without ROCK inhibitor. Bar chart represents the relative fold change in HK2 mRNA expression in each group (n= 3 independent experiments). Statistical tests were two-sided. Results are given as means ± SEM. (a-f) Student’s t test.

Supplementary figure 8

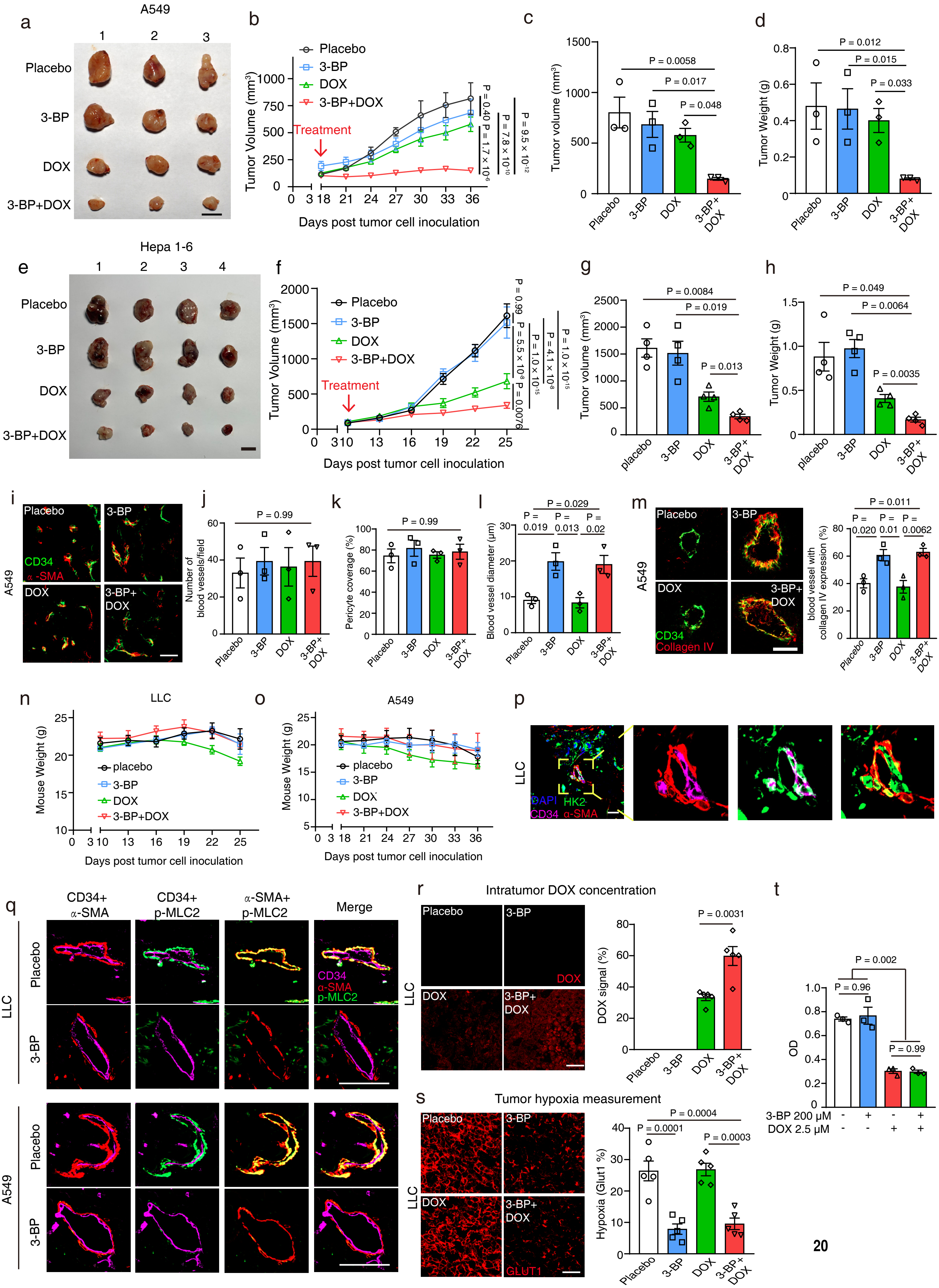

Supplementary fig. 8: Combination treatment of HK2 inhibitor and doxorubicin inhibits subcutaneous tumor growth while reducing tumor hypoxia. (a-h) Nude mice/C57/BLK6 mice were subcutaneously injected with either 3×10^6 A549 or Hepa 1-6 cells, which were then treated with either placebo, 3-BP, doxorubicin (DOX) alone or 3-BP and DOX combination. Representative gross tumor image from each group is given. (b, f) Tumor growth curve showed that combination treatment of HK2 inhibitor and doxorubicin strongly inhibited A549/Hepa 1-6 subcutaneous tumor growth as compared to placebo, 3-BP or DOX alone group. (c, d, g, h) Bar charts show the tumor volume and weight of each treatment group (n=3 independent animals per group (a-d) and n=4 biologically independent animals per group (e-h)). (i) Tumor sections double-immunostained for α-SMA (red) and CD34 (green). (j) Mean blood vessel number per field. (k) Mean percentage of pericyte coverage. (l) Mean tumor blood vessel diameter. (m) Tumor sections double-immunostained for collagen IV (red) and CD34 (green). (n, o) The body weight of LLC/A549 tumor bearing mice (n=5 biologically independent animals per group (n); n=3 biologically independent animals per group (o)). (p) Triple immunostaining of CD34 (magenta), HK2 (green) and α-SMA (red) in LLC tumor section (n=3 independent animals per group (i-m, p)). (q) Representative images of CD34 (magenta), p-MLC2 (green) and α-SMA (red) immunostained tumor sections derived from placebo or 3-BP treated mice (n=3 independent animals per group). (r) Quantification of LLC intratumoral doxorubicin intensity by confocal microscopy imaging (n=5 independent animals per group). (s) Representative immunostaining images of GLUT1 in LLC tumor sections derived from each treatment group. Bar charts show the mean percentage of GLUT1 staining in each group (n=5 independent animals per group). (t) LLC cells were treated with either 200 μM 3-BP, 2.5 μM doxorubicin or 3-BP and doxorubicin combination for 48 hours (n=3 independent experiments). Results are given as means ± SEM. (b, f) Two-way ANOVA. (c, d, g, h, j-m, s, t) One-way ANOVA. (r) Student's t test. Scale bars in (a, e) represents 1 cm, (i) 50 μm, (m) 20 μm,

(p, q) 25 μm, (r, s) 50 μm.

Supplementary figure 9

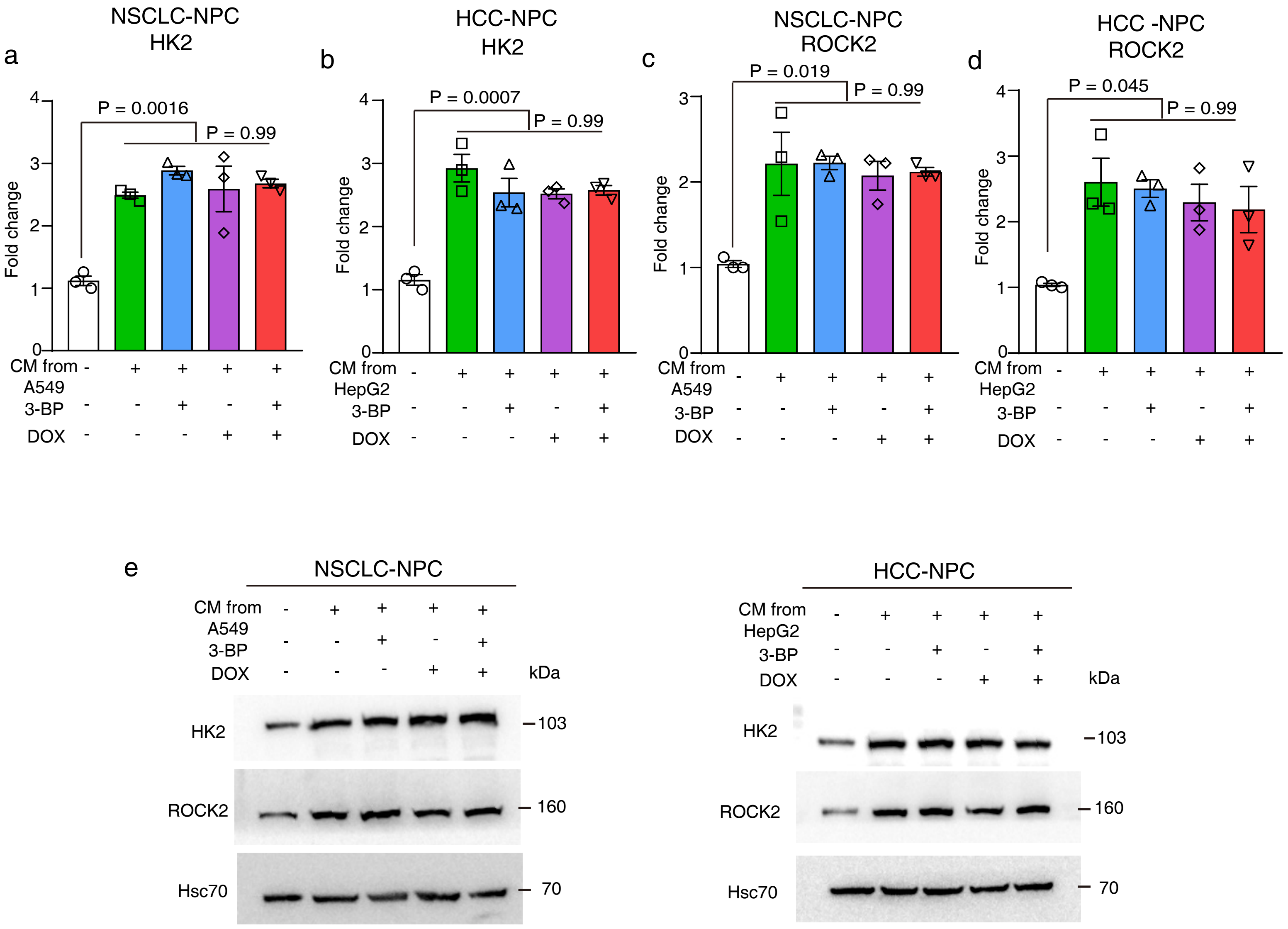

Supplementary fig. 9: Exposure of NPC with conditioned medium from HK2 inhibitor or/and doxorubicin pre-treated cancer cells can still up-regulate pericyte-HK2 and ROCK2 expression as compared with untreated NPC. (a-e) RT-PCR and Western blot analysis of the expression of HK2 and ROCK2 in NSCLC/HCC derived NPC after exposed or not exposed with conditioned medium from either doxorubicin or/and 3-BP pre-treated or untreated A549/HepG2 cells. Bar charts represent the relative fold change in HK2/ROCK2 mRNA expression in each group as indicated (n=3 experimental repeats). Results are given as means ± SEM. (a-d) One-way ANOVA.

Supplementary figure 10

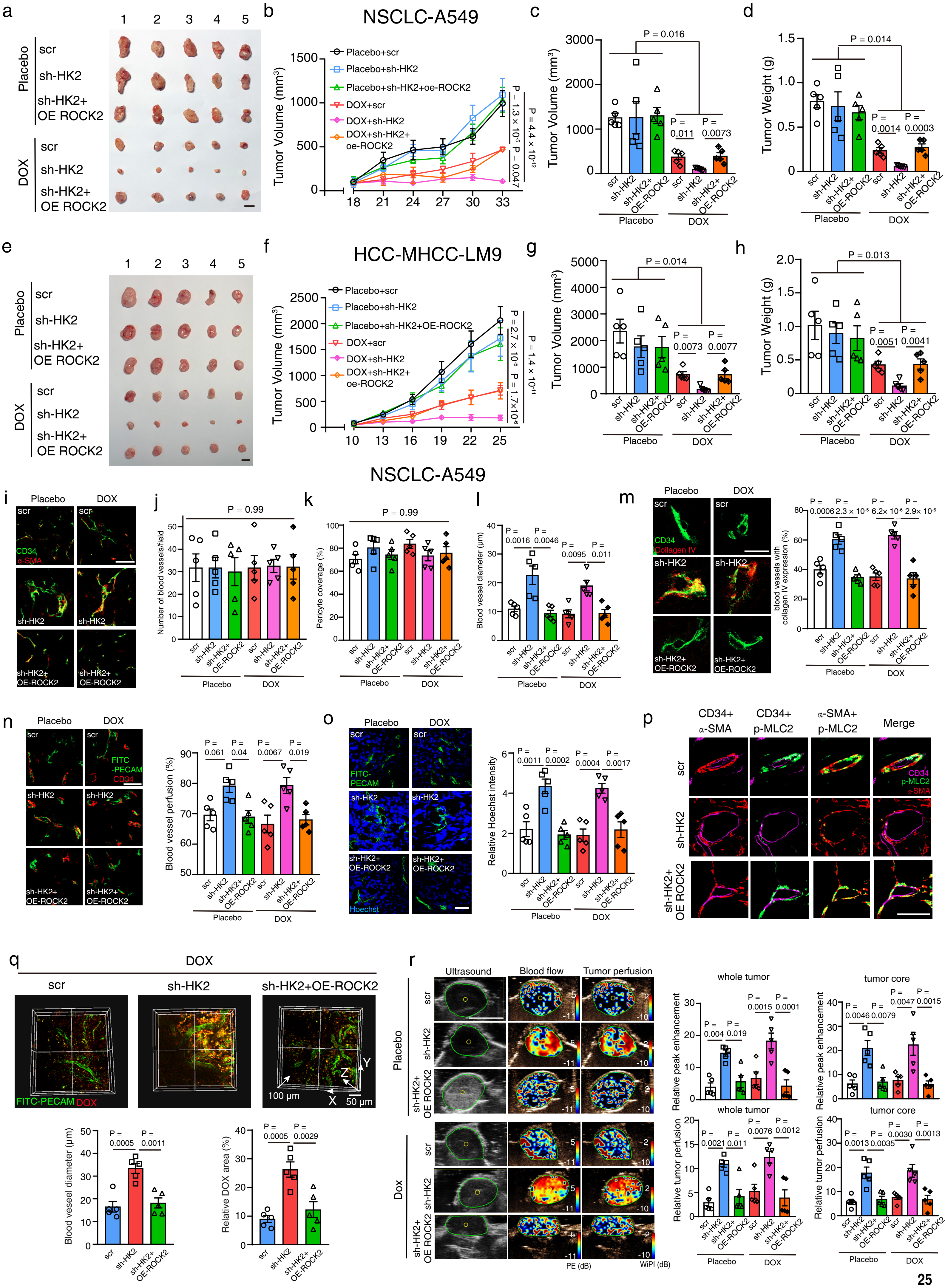

Supplementary fig. 10: Depletion of pericyte-HK2 expression enhances doxorubicin efficacy against tumor growth. (a-h) Nude mice were subcutaneously co-injected with A549/MHCC-LM9 cells and indicated TPC (10:1 ratio). The tumor bearing mice were then treated with either placebo or doxorubicin (DOX). (b, f) Tumor growth curve showed that depletion of pericyte-HK2 enhanced doxorubicin efficacy against A549 and MHCC-LM9 tumor growth as compared with scramble control group, while overexpression of ROCK2 in HK2-depleted TPC reduced the enhanced effect observed. (c, d, g, h) Bar charts show the tumor volume and weight of each group (n= 5 mice per group). (i) Tumor sections were double immunostained for α-SMA (red) and CD34 (green). (j) Mean tumor blood vessel diameter, (k) Mean percentage of pericyte coverage, (l) Mean tumor blood vessel diameter, (m) Tumor sections double-immunostained for collagen IV (red) and CD34 (green) (n=5 animals per group (i-m)). (n) Representative of FITC-PECAM perfused tumor blood vessels with CD34 immunostaining (n=5 tumors per group). (o) Bar charts show the relative area of Hoechst dye uptake into perivascular tumor cells to area of FITC-PECAM in each group (n=5 animals per group). (p) Representative images of CD34 (magenta), p-MLC2 (green) and α-SMA (red) immunostained tumor sections are given from indicated groups (n=5 animals per group). (q) *Ex vivo* two photon microscopic imaging of doxorubicin treated tumors arising from the mice that were co-injected with A549 and either scramble transfected TPC, HK2-depleted TPC or ROCK2 overexpressing HK2-depleted TPC. Bar charts show the quantification of vessel diameter and doxorubicin in each group (n=5 animals per group). (r) Doppler ultrasonography in live mice provides relative blood flow (peak enhancement, PE) and tumor perfusion (Wash-in perfusion index, WiPI) in subcutaneous tumors derived from co-injection of A549 and indicated TPC, after treated with placebo or doxorubicin. Representative ultrasound images (n=5 animals per group). Bar charts, quantitation across the whole tumors, including tumor cores. Results are given as means ± SEM. (b, f) Two-way ANNOVA. (c, d, g, h, j-r) One-way ANNOVA. Scale bars in (a, e, r)

represent 1 cm, (i, n, o, q) 50 μm, (m, p) 20 μm.
