# Supplementary material for: Hexokinase 2-driven glycolysis in pericytes activates their contractility leading to tumor blood vessel abnormalities
Source: Nat Commun. 2021 Oct 14;12:6011. doi: 10.1038/s41467-021-26259-y (PMC8517026; doi:10.1038/s41467-021-26259-y)
Supplement: Supplementary file 3 — Description of Additional Supplementary Files [file 41467_2021_26259_MOESM3_ESM.pdf]

Description of Additional Supplementary Files

Supplementary Movie 1: Ex vivo two photon microscopic imaging of FITC-PECAM antibody perfused LLC tumors after treated with DOX alone.

Supplementary Movie 2: Ex vivo two photon microscopic imaging of FITC-PECAM antibody perfused LLC tumors after treated with 3-BP and DOX combination.

Supplementary Movie 3: Ex vivo two photon microscopic imaging of doxorubicin treated tumors arising from the mice that were co-injected with A549 and scramble transfected TPC.

Supplementary Movie 4: Ex vivo two photon microscopic imaging of doxorubicin treated tumors arising from the mice that were co-injected with A549 and HK2-depleted TPC.

Supplementary Movie 5: Ex vivo two photon microscopic imaging of doxorubicin treated tumors arising from the mice that were co-injected with A549 and ROCK2 overexpressing HK2- depleted TPC.
