# Supplementary material for: Hexokinase 2-driven glycolysis in pericytes activates their contractility leading to tumor blood vessel abnormalities
Source: Nat Commun. 2021 Oct 14;12:6011. doi: 10.1038/s41467-021-26259-y (PMC8517026; doi:10.1038/s41467-021-26259-y)
Supplement: Supplementary file 9 — Reporting summary [file 41467_2021_26259_MOESM9_ESM.pdf]

nature research

Corresponding author(s): Ping-Pui Wong

Last updated by author(s): Sep 5, 2021

Reporting Summary

Nature Research wishes to improve the reproducibility of the work that we publish. This form provides structure for consistency and transparency in reporting. For further information on Nature Research policies, see our Editorial Policies and the Editorial Policy Checklist.

Statistics

For all statistical analyses, confirm that the following items are present in the figure legend, table legend, main text, or Methods section.

n/a	Confirmed	
☐	☒	The exact sample size (n) for each experimental group/condition, given as a discrete number and unit of measurement
☐	☒	A statement on whether measurements were taken from distinct samples or whether the same sample was measured repeatedly
☐	☒	The statistical test(s) used AND whether they are one- or two-sided<br>*Only common tests should be described solely by name; describe more complex techniques in the Methods section.*
☐	☒	A description of all covariates tested
☐	☒	A description of any assumptions or corrections, such as tests of normality and adjustment for multiple comparisons
☐	☒	A full description of the statistical parameters including central tendency (e.g. means) or other basic estimates (e.g. regression coefficient) AND variation (e.g. standard deviation) or associated estimates of uncertainty (e.g. confidence intervals)
☐	☒	For null hypothesis testing, the test statistic (e.g. F, t, r) with confidence intervals, effect sizes, degrees of freedom and P value noted<br>*Give P values as exact values whenever suitable.*
☒	☐	For Bayesian analysis, information on the choice of priors and Markov chain Monte Carlo settings
☐	☒	For hierarchical and complex designs, identification of the appropriate level for tests and full reporting of outcomes
☒	☐	Estimates of effect sizes (e.g. Cohen's d, Pearson's r), indicating how they were calculated

Our web collection on statistics for biologists contains articles on many of the points above.

Software and code

Policy information about availability of computer code

Data collection	-All images were obtained by microscopy (Zeiss LSM 800 with airyscan, Nikon NI-U, Olympus IX71, MD ImageXpress Micro Confocal, Olympus FVMPE-RS).<br>-FACS data were obtained by flow cytometry (Beckman CytoFLEX, Beckman MoFlo EQs).<br>-RT-PCR data were obtained by Roche LightCycler 480 II.<br>-The extracellular acidification rate (ECAR) and oxygen consumption rate (OCR) were obtained by Seahorse XFe96 .<br>-The data of metabolic flux were obtained by Thermo 1300.<br>-Tumor perfusion data were obtained by Vevo® 2100 system with a MS250 transducer.<br>-Optical density data were obtained by TECAN Spark10M.<br>-Proteomics data were obtained by Orbitrap Exploris 480 System with Ion Max Source.<br>-Western Blot data were obtained by Mini Chemi 610.
Data analysis	-Carl Zeiss ZEN 2011 (black edition)<br>-ImageJ version2.1.0<br>-Microsoft Excel version 365<br>-Graphpad Prism 8.0.1<br>-cytoExpert 2.0<br>-FlowJo v10<br>-LightCycler480 1.5.1<br>-Kaplan-Meier plotter(https://kmplot.com/analysis/)<br>-Tracefinder 4.1 (Thermo Scientific)<br>-Proteome Discoverer software suite (v2.3, Thermo Fisher Scientific)<br>-FV31S-SW (v1.0)

April 2020

-VevoCQ contrast quantification software (Vevo LAB 3.1.1)
-Wave (v2.3)
-Sage Capture (v1.2)

For manuscripts utilizing custom algorithms or software that are central to the research but not yet described in published literature, software must be made available to editors and reviewers. We strongly encourage code deposition in a community repository (e.g. GitHub). See the Nature Research guidelines for submitting code & software for further information.

Data

Policy information about availability of data

All manuscripts must include a data availability statement. This statement should provide the following information, where applicable:

- Accession codes, unique identifiers, or web links for publicly available datasets
- A list of figures that have associated raw data
- A description of any restrictions on data availability

The mass spectrometry proteomics data generated in this study has been deposited to the Proteome X consortium via the PRIDE partner repository with the dataset identifier PXD026963. The source data underlying Figs. 1c, f, k, l, 2c-g, 3a, b, d-f, h-k, 4b, d-k, 5b-d, f-k, m-p, r-t, 6b-d, f-k, m, n, p and Supplementary Figs. 3c-l, 4b-d, f, 5a-e, 6a-d, f-k, m-o, q-w, 7a-g, 8b-d, f-h, j-o, r-t, 9a-e, 10b-d, f-h, j-o, q, r are provided as source data. The use of publicly available data from NSCLC and HCC were consulted on the websites: https://kmplot.com/analysis/index.php?p=service&cancer=lung; https://kmplot.com/analysis/index.php?p=service&cancer=pancancer_rnaseq), under the specific product names: KM Plotter-Lung Cancer and-Pan-cancer RNA-seq. All other relevant data supporting the key findings of this study are available within the article and its supplementary information files or from the corresponding author upon reasonable request. A reporting summary for this article is available as a supplementary information file. Source data are provided within this paper.

Field-specific reporting

Please select the one below that is the best fit for your research. If you are not sure, read the appropriate sections before making your selection.

[x] Life sciences [ ] Behavioural & social sciences [ ] Ecological, evolutionary & environmental sciences

For a reference copy of the document with all sections, see nature.com/documents/nr-reporting-summary-flat.pdf

Life sciences study design

All studies must disclose on these points even when the disclosure is negative.

Sample size	The sample size of the Kmplotter database was determined by the number of tumor samples analysed with RNA sequencing. Sample size and number of independent experiments are stated in the figure legend. Three to more independent samples/experiments were used to perform statistical analysis. For our own patient cohorts, sample size was not statistically determined before collection. The determination of sample size is based on our experience and numerous publications, which is sufficient to generate statistically significant results.
Data exclusions	No data were excluded.
Replication	Each experiment was repeated at least 3 times independently, unless stated otherwise. The exact number of replicates were included in the legend or method section.
Randomization	Animal with similar ages and weight were randomly allocated to experimental groups before treated with corresponding drug combination or placebo. For experiments other than animals, the samples/cells were randomly allocated into different groups prior to treatment.
Blinding	For microscopy, flow cytometry, and other data collected by objective instruments, the researchers were not blinded to group allocation because they need to know which group each raw data corresponds to. But they were blinded during data analysis. The researchers who performed animal experiments was not blinded because they needed to know how to treat mice with different drug combination or placebo. But the researchers were blinded during the data analysis.

Reporting for specific materials, systems and methods

We require information from authors about some types of materials, experimental systems and methods used in many studies. Here, indicate whether each material, system or method listed is relevant to your study. If you are not sure if a list item applies to your research, read the appropriate section before selecting a response.

Materials & experimental systems

n/a	Involved in the study	
☐	☒	Antibodies
☐	☒	Eukaryotic cell lines
☒	☐	Palaeontology and archaeology
☐	☒	Animals and other organisms
☐	☒	Human research participants
☒	☐	Clinical data
☒	☐	Dual use research of concern

Methods

n/a	Involved in the study	
☒	☐	ChIP-seq
☐	☒	Flow cytometry
☒	☐	MRI-based neuroimaging

Antibodies

Antibodies used

-mouse mAb against FITC-conjugated anti-human CD146 antibody (Biolegend, 361012), 1:100
-mouse mAb against PE-conjugated anti-human CD45 (Biolegend, 368510), 1:100
-mouse mAb against PE/Cy7-conjugated anti-human CD31 (Biolegend, 303118), 1:100
-mouse mAb against APC-conjugated anti-human CD140b (PDGFRβ) (Biolegend, 323608), 1:100
-mouse mAb against BV421-conjugated anti-human CD34 (Biolegend, 343610),1:100
-fixable viability dye (FVD) eFluor® 780 (ebioscience, 65-0865-14), 1:300
-mouse mAb against APC-conjugated anti-human CD13 (Biolegend, 301706),1:300
-rabbit mAb against human CD34 (ZSGB-BIO, ZM-0046), 1:300
-rabbit mAb against human/mouse CD34 (Abcam, ab81289), 1:300
-mouse mAb against human CD146 (ZSGB-BIO, ZM-0299), 1:100
-mouse mAb against human/mouse α-smooth muscle-Cy3TM antibody (Sigma, C6198), 1:500
-rabbit mAb against human PDGFRβ (CST, 3169S), 1:100
-rabbit mAb against human/mouse Glut1 (Abcam, ab652), 1:100
-rabbit mAb against human/mouse Hexokinase II (Abcam, ab227198), 1:100
-rabbit mAb against human/mouse ROCK2 (Merck, HPA007459), 1:100
-rabbit mAb against human ROCK1 (CST, 4035),1:1000
-rabbit mAb against human/mouse p-MLC2 (CST, 3671S), 1:1000
-rabbit mAb against human FAP (E1V9V) (CST, 66562), 1:100
-mouse mAb against human desmin (Merck Millipore, MA1:100B3430), 1:100
-rabbit mAb against human collagen IV (Abcam, ab6586), 1:100
-Rabbit mAb against human/mouse ROCK2 (CST, 8236S), 1:1000
-mouse mAb against human Hsc70 was used as loading control (Santa Cruz, sc-7298), 1:1000
-rat monoclonal anti mouse -FITC -PECAM (Biolegend, 102406), 100ul
-mouse mAb against PE anti-human CD44 Antibody (Biolegend, 338808)1:100
-donkey mAb against mouse or rabbit Alexa Fluor–conjugated secondary antibodies (Invitrogen Molecular Probes, A21202, A21206,, A31570, A31572 and A21082) diluted 1:1000
-goat mAb against mouse or rabbit horseradish peroxidase (HRP)-conjugated antibody (CST, 7074S or 7076S) diluted 1:1000

Validation

The antibody validation information corresponding to above are as following:
-https://www.biolegend.com/en-us/products/fitc-anti-human-cd146-antibody-9277
-https://www.biolegend.com/en-us/products/pe-anti-human-cd45-antibody-12396
-https://www.biolegend.com/en-us/products/pe-cyanine7-anti-human-cd31-antibody-6124
-https://www.biolegend.com/en-us/products/apc-anti-human-cd140b-pdgfrbeta-antibody-4054
-https://www.thermofisher.com/order/catalog/product/65-0865-14#/65-0865-14
-https://www.biolegend.com/en-us/products/apc-anti-human-cd13-antibody-874
-http://www.zsbio.com/product/ZM-0046
-https://www.abcam.com/cd34-antibody-ep373y-ab81289.html
-http://www.zsbio.com/product/ZM-0299
-https://www.sigmaaldrich.com/catalog/product/sigma/c6198?lang=zh&region=CN
-https://www.cellsignal.com/products/primary-antibodies/pdgf-receptor-b-28e1-rabbit-mab/3169?Ntk=Products&Ntt=3169&productId=5597
-https://www.abcam.com/glucose-transporter-glut1-antibody-ab652.html
-https://www.abcam.com/hexokinase-ii-antibody-ab227198.html
-https://www.sigmaaldrich.com/catalog/product/sigma/hpa007459?lang=zh&region=CN
-https://www.cellsignal.com/products/primary-antibodies/phospho-myosin-light-chain-2-ser19-antibody/3671?Ntk=Products&Ntt=3671
-https://www.abcam.com/cd13-antibody-epr4058-ab108310.html
-https://www.emdmillipore.com/US/en/product/Anti-Desmin-Antibody-clone-DE-B-5,MM_NF-MAB3430
-https://www.abcam.com/collagen-iv-antibody-ab6586.html
-https://www.cellsignal.com/products/primary-antibodies/rock2-antibody/8236?Ntk=Products&Ntt=8236
-https://www.scbt.com/zh/p/hsc-70-antibody-b-6
-https://www.biolegend.com/en-us/products/fitc-anti-mouse-cd31-antibody-120
-https://www.cellsignal.com/products/primary-antibodies/fap-e1v9v-rabbit-mab/66562

-https://www.cellsignal.com/products/primary-antibodies/rock1-c8f7-rabbit-mab/4035
-https://www.biolegend.com/en-us/search-results/brilliant-violet-421-anti-human-cd34-antibody-7708
-https://www.biolegend.com/en-us/products/pe-anti-human-cd44-antibody-5745
-https://www.thermofisher.cn/search/results?query=Donkey%20anti-Mouse%20IgG%20(H+L)&focusarea
-https://www.cellsignal.com/products/secondary-antibodies/anti-rabbit-igg-hrp-linked-antibody/7074?Ntk=Products&Ntt=7074
-https://www.cellsignal.com/products/secondary-antibodies/anti-rabbit-igg-hrp-linked-antibody/7074?Ntk=Products&Ntt=7076

Eukaryotic cell lines

Policy information about cell lines

Cell line source(s)	-Human umbilical vein endothelial cells (HUVEC) were isolated from human umbilical cord.<br>-Human primary dermal fibroblasts (HFF-1, SCSP-109) were purchased from National Infrastructure of Cell Line Resource .<br>-Human normal adjacent tissue derived pericytes and tumor derived pericytes were isolated from human NSCLC and HCC tissues.<br>-Peripheral blood mononuclear cells (PBMC) were isolated from buffy coats derived from the blood of healthy donors.<br>-Human primary pulmonary artery smooth muscle cells (SMC, PCS-100-023) were purchased from ATCC.<br>-Lewis lung carcinomas (LLC), A549 and HepG2 cells (all from ATCC) .<br>-LM9 cells were given by professor Zhuang Shimei.
Authentication	Cell lines were used as provided commercially and no additional identification was performed.
Mycoplasma contamination	No mycoplasma contamination was detected during the program.
Commonly misidentified lines (See ICLAC register)	No commonly misidentified lines were used in the project.

Animals and other organisms

Policy information about studies involving animals; ARRIVE guidelines recommended for reporting animal research

Laboratory animals	4-6 weeks old female C57/BLk6 and nude mice purchased from the Guangdong Medical Science Experiment Center were used in this study. Mice were allowed to acclimate to local conditions for 1 week and housed at 22 ±2 celcius degree, humidity 50 ±10% under a 12h dark/12h light cycle with adequate food and water.
Wild animals	No wild animals were used in this study.
Field-collected samples	No field-collected samples were used in this study.
Ethics oversight	All animal procedures were approved by the Institutional Animal Care and Use Committee (IACUC) of Sun Yat-sen University.

Note that full information on the approval of the study protocol must also be provided in the manuscript.

Human research participants

Policy information about studies involving human research participants

Population characteristics	Human non-small cell lung cancer (n= 10 paired normal adjacent tissues and tumor tissues; n= 77 patients for survival study) and hepatocellular carcinoma paraffin embedded samples (n= 10 paired normal adjacent tissues and tumor tissues; n =101 patients for survival study) were obtained from Sun Yat-sen Memorial hospital (Guangzhou, China) with complete clinical data. The NSCLC patients and HCC patients who underwent curative freshly resected tissue enrolled for pericyte isolation were based on the following main criteria: (1) patients were absence of anticancer therapies prior to the operation. (2) No concurrent autoimmune disease, HIV, or syphilis.
Recruitment	Clinical samples were collected from Sun Yat-sen Memorial Hospital (Guangzhou, China) with complete clinical data. The bio-specimens from each individual were collected at the time of surgery.
Ethics oversight	This study was approved by the Institutional Review Board of Sun Yat-sen Memorial Hospital (Guangzhou, China).

Note that full information on the approval of the study protocol must also be provided in the manuscript.

Flow Cytometry

Plots

Confirm that:

☒ The axis labels state the marker and fluorochrome used (e.g. CD4-FITC).

☒ The axis scales are clearly visible. Include numbers along axes only for bottom left plot of group (a 'group' is an analysis of identical markers).

☒ All plots are contour plots with outliers or pseudocolor plots.

☒ A numerical value for number of cells or percentage (with statistics) is provided.

Methodology

Sample preparation	Sample preparation steps are described in the 4th part of MATERIALS AND METHODS section.
Instrument	Beckman CytoFLEX, Beckman MoFlo EQs
Software	CytoExpert 2.0, FlowJo V10
Cell population abundance	Post-sorted cells were analyzed by flow cytometry and the purity was shown in Figure 2A and Supplementary figure 2A
Gating strategy	The gating strategy are described in Figure 1E-H and Supplementary figure 1 B-E

☒ Tick this box to confirm that a figure exemplifying the gating strategy is provided in the Supplementary Information.
